# Case of Pernicious Anæmia

**Published:** 1886-12

**Authors:** Thomas Cole

**Affiliations:** Physician to the Royal United Hospital, Bath.


### Clinical Records.

---

CASE OF PERNICIOUS ANÆMIA. By THOMAS COLE, M.D. Lond., M.R.C.P., Physician to the Royal United Hospital, Bath.

W. B., a cabman, æt. 35, was admitted into the Royal United Hospital, Bath, on June 21st, 1886.

He had always lived in England. He had acute rheumatism seven years ago. His health had failed since January—dyspnœa, nocturnal cough, and anorexia being the prominent symptoms. He was extremely pale, and had a troublesome hacking cough, with very short breath on exertion. There was dulness at the right apex, with defective respiratory murmur and crepitation. There was a double cardiac bruit audible at base and apex, and loudest over the sternum. The liver and spleen were much enlarged. The feet and legs were œdematous. The urine contained no albumen, and its density was normal. His pulse was 96. The temperature was invariably pyrexial in the evening for a considerable period, but gradually became normal. There were numerous variously shaped retinal hæmorrhages, mostly striate, and radiating from the optic discs. The red blood-cells averaged about 940,000 per cubic mm. of blood, and the white about 7500. He expectorated some blood-stained sputum. He was placed upon tartrate of iron, cream of tartar, and digitalis. Under this treatment the œdema subsided, and the general health improved. But the liver and spleen increased in size, and the pallor was extreme. He also had some epistaxis. On July 5th there was a trace of albumen in the urine. On June 28th he was put upon hydrochloric acid, sulphate of iron grs. ii., and liq. arsen. hydrochlor. ♏ iii. ter die. The quantity of arsenic was gradually increased till, on August 2nd, a dose of ♏ xii. t.d. was attained. On July 19th the spleen had subsided, and the liver was smaller. On the 26th the red blood-cells had increased to 1,780,000 per cub. mm. On August 3rd the weight was 10 st. 2 lbs., the cough had gone, the lung trouble seemed obsolete, and the œdema had almost disappeared. Once only was there a trace of albumen. On August 7th he was discharged, fairly well. He lately presented himself at the hospital in good health, all the morbid symptoms having ceased.

That this was a case of pernicious anæmia cannot be doubted. It is not the first case which I have seen recover. There is a ring of despair about the word "pernicious" which, however useful as indicative of the serious nature of the malady, may be injurious by inducing a hopeless attitude in the practitioner's mind, by no means conducive to a patient and persistent attack on so powerful an enemy. The symptomatology of this disease is certainly suggestive of some species of blood-infection, and very possibly an external influence may one day be detected as its cause. Perhaps this may be the reason why arsenic is so useful in this and other forms of anæmia. Be this as it may, it was unquestionably most valuable in the case in question; and I should, without hesitation, recommend the administration of much larger doses of the drug, if necessary, in any similar cases.

